# Marine Rare Actinomycetes: A Promising Source of Structurally Diverse and Unique Novel Natural Products

**DOI:** 10.3390/md17050249

**Published:** 2019-04-26

**Authors:** Ramesh Subramani, Detmer Sipkema

**Affiliations:** 1 School of Biological and Chemical Sciences, Faculty of Science, Technology & Environment, The University of the South Pacific, Laucala Campus, Private Mail Bag, Suva, Republic of Fiji; subramani_r@usp.ac.fj; 2 Laboratory of Microbiology, Wageningen University & Research, Stippeneng 4, 6708 WE Wageningen, The Netherlands

**Keywords:** rare actinomycetes, marine actinobacteria, cultivation, natural products, bioactive compounds

## Abstract

Rare actinomycetes are prolific in the marine environment; however, knowledge about their diversity, distribution and biochemistry is limited. Marine rare actinomycetes represent a rather untapped source of chemically diverse secondary metabolites and novel bioactive compounds. In this review, we aim to summarize the present knowledge on the isolation, diversity, distribution and natural product discovery of marine rare actinomycetes reported from mid-2013 to 2017. A total of 97 new species, representing 9 novel genera and belonging to 27 families of marine rare actinomycetes have been reported, with the highest numbers of novel isolates from the families Pseudonocardiaceae, Demequinaceae, Micromonosporaceae and Nocardioidaceae. Additionally, this study reviewed 167 new bioactive compounds produced by 58 different rare actinomycete species representing 24 genera. Most of the compounds produced by the marine rare actinomycetes present antibacterial, antifungal, antiparasitic, anticancer or antimalarial activities. The highest numbers of natural products were derived from the genera *Nocardiopsis*, *Micromonospora*, *Salinispora* and *Pseudonocardia*. Members of the genus *Micromonospora* were revealed to be the richest source of chemically diverse and unique bioactive natural products.

## 1. Introduction

Emerging infectious diseases and multidrug-resistant human pathogens are becoming a major threat to global health [1]. Therefore, there is an urgent need for new antibiotics to fight evolving bacterial infections. Despite the use of large synthetic combinatorial libraries of molecules to develop novel drugs, natural products and microbial metabolites, in particular, are a predominant source of bioactive scaffolds that represent the foundation for the development of life-saving antibiotics [2]. Nature encompasses millions of prokaryotes and eukaryotes with particularly high diversity in oceans and rainforests. However, so far only a small fraction (approximately 250,000–300,000 living species) of at least 1.5 million fungi, 0.5 million plant species and 10^11^–10^12^ microbial species on Earth have been documented [3,4]. Moreover, even the known species have only been explored for bioactivity or for natural product discovery up to a limited extent. Therefore, natural resources are virtually unlimited for natural product discovery. The phylum Actinobacteria represents one of the largest phyla among the 30 major phyla currently recognized within the domain Bacteria. There are 6 classes, 18 orders, 14 suborders, 63 families and 374 genera recorded in this phylum until October 2016 (http://www.bacterio.net/-classifphyla.html#actinobacteria). In this review paper the term “actinomycetes” (http://www.bacterio.net/-classifphyla.html#actinobacteria) will be used to refer the members of the order Actinomycetales of the phylum Actinobacteria. The members of actinomycetes have been characterized as the most important group of microorganisms in the field of biotechnology, as producers of bioactive secondary metabolites with medical, industrial and agricultural applications [5]. However, until now, only less than 1% of the actinomycetes have been identified, investigated and documented [3]. Out of 500,000 natural compounds reported worldwide from biological sources, approximately 70,000 are microbially-derived compounds (both from bacteria and fungi), of which 29% is derived from actinomycetes. Approximately 60% of antibiotics applied, were isolated from actinomycetes between 1950 and 1970, exclusively from the genus *Streptomyces* [3]. However, in more recent history, high replication of discovery of compounds has been reported from *Streptomyces* species, which diverted the attention to non-*Streptomyces* actinomycetes and a noteworthy renaissance in antibiotics development from microorganisms has come with the exploration of previously poorly assessed microorganisms from underexplored environments. The unexplored and underexplored environments including marine ecosystems are promising sources of rare actinomycetes that are believed to be rich sources of interestingly new compounds [6,7]. ‘Rare actinomycetes’ are defined as the actinomycete strains less frequently isolated than that of the ‘commonly’ isolated *Streptomyces* spp., even though they may not actually be rare in the environment.

Oceans occupy 71% of the Earth’s surface holding 97% of the planet’s water and nearly 87% of life with essentially untouched fauna and flora [3] and are a great source for undiscovered organisms including microorganisms and novel natural products. Marine-derived rare actinomycetes are reported to be a potentially rich source of diverse chemicals, structurally unique secondary metabolites and novel therapeutic compounds [2,6]. Only 11 rare actinomycetes genera had been reported by 1970, followed by 100 genera by 2005 and 220 genera by 2010 [7]. High-throughput metagenome sequencing methods have expanded our knowledge and revealed the presence of many novel actinomycetes that were not previously detected in cultivation studies [5,8,9]. The retrieval of rare actinomycetes in conventional cultivation experiments is generally lower than that of the streptomycete strains [5]. However, recent understanding of marine actinomycetes’ physiological, chemical, and structural features has enabled the design of selective isolation media [5]. A total of 13,700 bioactive metabolites were reported from actinomycetes up to 2010, of which 10,400 were derived from streptomycetes and 3300 from rare actinomycete strains [3]. In 1974, only 125 active metabolites had been isolated from rare actinomycetes, increasing to 361, 745, 1276, 2250, 2500 bioactive metabolites by 1980, 1984, 1988, 2005 and 2010, respectively [7]. In our previous review, we summarized the novel families, novel genera, and new species of rare actinomycetes from marine habitats including bioactive compounds reported from 2007 to mid-2013 [7]. The goal of this present review is to summarize new species of marine rare actinomycetes, and the bioactive compounds discovered between mid-2013 and 2017 and discuss their chemical diversity and biotechnological potential.

## 2. Isolation Methods for Marine Rare Actinomycetes

Members of the phylum Actinobacteria adapt well to and successfully colonize different extreme environments including the deep sea [10] and genera of this phylum exhibit huge diversity in terms of their morphology, physiology, and metabolic capabilities [5]. Marine rare actinomycetes generally require special growth conditions compared to terrestrial actinomycete species [11,12,13,14]. Notably, it has been observed that a large number of bacterial cells in under/unexplored environments are viable but not culturable (VBNC), as approximately 1% of bacterial cells can form colonies on isolation media by conventional methods [15]. Therefore, high throughput molecular techniques, including metagenomics, are increasingly favored to investigate microbial communities in the environment [16] for which culture-based approaches have been rather unsuccessful up to now. Concurrently, knowledge of functional characteristics of actinomycetes based on cultivation-independent studies, has led to improved strategies with respect to growth conditions and cultivation media to recover previously unculturable actinomycetes [5,17,18,19,20,21].

### 2.1. Basic Approaches for Isolation Media for Marine Rare Actinomycetes

Targeting unknown rare actinomycetes for isolation requires knowledge and experience of actinomycetes taxonomy, physiology and environmental factors, such as pH, cultivation temperature, oxygen, nutrient requirements etc. [22]. Sodium, is one of the most important medium components for growth of marine microorganisms including marine actinomycetes such as *Salinispora* spp. and therfore growth media should generally have osmotic values similar to seawater [13]. Besides, different carbon (soluble starch, glucose, dextrose, maltose, trehalose, mannitol, raffinose, fucose, chitin, glycerol and oatmeal) and combined carbon-nitrogen sources (peptone, yeast extract, casein, malt extract, meat extract, beef extract and tryptone) have been supplemented in isolation media for successful isolation of marine rare actinomycete taxa [23,24,25,26,27]. In addition, researchers have added sediment extracts, sponge extracts and natural seawater alone or as a supplement to mimic natural environmental conditions [12,28,29,30,31,32,33,34]. In general, low-nutrient media are more efficient than nutrient-rich media for isolation of marine rare actinomycetes [13,28,35]. Generally, some basic approaches may be followed for isolation of marine rare actinomycetes: 1, Three to five different isolation media with various components should be employed for any target genus of actinomycetes [9,11,28]; 2, The isolation media must meet the requirements of the target actinomycetes and at the same time should limit the growth of unwanted microbes [22,28]; 3, Growth inhibitors, in the form of antibiotics or chemicals, should be added into isolation media to inhibit or restrict the growth of Gram-negative bacteria and fungi [11,28]; 4, The medium should be so designed that it mimicks the microbe’s natural environmental conditions [12,28,29]; 5, The medium should also suppress the growth of fast-growing and common streptomycete strains [22].

### 2.2. Pretreatment of Marine Samples 

Marine samples, particularly sediments used for the isolation of rare actinomycetes, may be treated prior to isolation to remove common terrestrial actinomycetes and unwanted microorganisms to reduce replication of isolation. Commonly used pre-treatment methods for the isolation of rare actinomycetes from marine samples generally include dilution and mixing with sterile natural seawater [25,36,37], artificial seawater [38,39,40], deionized/distilled water supplemented with NaCl [41,42], multi-salts [24,26,40], vitamin B mixtures [43], one-quarter Ringer’s solution [44] and saline solution [45] before transferring the inoculum to Petri dishes [44]. A variety of pre-treatment methods for selectively isolating actinomycetes has been applied (Table 1). However, the drying of the environmental sample using laminar air flow, dilution with seawater or saline prior to sample heating are most frequently employed pre-treatments (Table 1). Actinomycetes spores are generally resistant to desiccation and heating and can thus be used to select against other Gram positive bacteria [46]. Further, actinomycetes spores are resistant to a wide range of chemicals, such as benzethonium chloride, chlorhexidine gluconate, phenol, sodium dodecyl sulfate, and different antibiotics. These chemicals have been used to selectively isolate actinomycete taxa. Treatment with these chemicals for 30 min. can kill or inhibit aerobic Gram negative bacteria, endospore-forming bacilli and pseudomonads, thus increasing the chance of selectively isolating actinomycetes, and reduce other types of bacteria [22]. Additionally, ultrasonic waves can release actinomycetes propagules from sediment particles into suspension, thus also increasing the number of Actinobacterial strains and reducing unwanted bacteria [47].

## 3. Marine Habitats: The Largest Reservoir for Rare Actinomycetes 

The world’s oceans constitute more than 90% of the inhabitable space on the planet and it is the largest reservoir of life on Earth. Approximately 80% of all life on Earth lives in the ocean and the oceans harbour 32 out of 33 known animal phyla, of which 15 are exclusively marine [48].

Marine habitats are also a rich source of diverse and largely uncharacterized microbial communities including actinomycetes [62]. This habitat shows extreme variations in ecological pressure, including competition for space, predation, available nutrients, light, oxygen concentration and pressure. Marine organisms including actinomycetes have developed a diverse range of secondary metabolites with unique structural elements to ensure their survival in these habitats [63]. Diverse new rare species including novel genera and novel families of actinomycetes have been isolated from marine habitats, such as coastal, tidal and deep-sea sediments, marine organisms (sponges, corals and ascidians), seawater and also mangrove forests [7]. Approximately 220 genera of rare actinomycetes were reported from marine sources until 2010 [64] and in the following sections we summarize new rare actinomycete isolates from these habitats since then [7]. For this review we’ve applied a conservative threshold on labelling a species as “novel” when sharing less than 97% similarity of the 16S rRNA gene to known species [65,66,67,68,69,70]. For the labelling of genera and families as “novel” we followed Silva taxonomy [71].

### 3.1. Rare Actinomycetes from Marine Sediments, Seawater, Eukaryotic Hosts and Mangroves

Approximately 83% of marine sediments are more than 1000 m below sea level, so most marine sediments are located in a cold, lightless, high pressure habitat where food is supplied from distant surface waters [72]. Deep-sea environments are divided into three zones: the bathyal (depth range between 200 and 2000 m), the abyssal (depth from 2000 to 6000 m) and the hadal (depth below 6000 m) [73]. Especially the abyssal and hadal zones are largely unexplored. Highest biodiversity has been recorded at a depth of 3000 m and the heterogeneity of biomass is expanding to 5000 m [74,75]. Cold deep-sea muds have an astounding species richness and diversity compared to tropical rain forests [76]. The majority of these species has not been isolated in the laboratory and an estimated 95% of these species are unidentified and mostly considered as new species [74]. Actinomycetes, including rare actinomycetes, are abundant in diverse marine sediments. A total of 48 new rare actinomycete species belonging to 16 different actinomycete families were isolated from marine sediments in the period from mid 2013 to 2017 (Table 2). Among them, 5 novel genera: *Flaviflexus*, *Halopolyspora*, *Mariniluteicoccus*, *Sediminivirga* and *Haloactinomyces* were described. The actinomycete families reported from marine sediments to which the novel species belong are Pseudonocardiaceae (8 new species), Nocardioidaceae (5 new species), Nocardiopsaceae (4 new species), Microbacteriaceae (4 new species), Micrococcaceae (4 new species), Propionibacteriaceae (4 new species), Micromonosporaceae (3 new species), Nocardiaceae (2 new species), Demequinaceae (2 new species), Intrasporangiaceae (2 new species), Bogoriellaceae (2 new species), Acidimicrobiaceae (2 new species), Brevibacteriaceae (2 new species), Actinopolysporaceae (2 new species), Actinomycetaceae (1 new species) and Cellulomonadaceae (1 new species).

Although earlier culture-dependent studies have described microbial population size to be only a few hundred cells per mL of seawater, the staining of cells using fluorescence microscopy studies demonstrated nominal cell densities of >10^5^ cells per mL of seawater [77,78], which anticipates that the ocean harbors 3.6 × 10^29^ microbial cells [79]. The microorganisms in the seawater play an important role in marine biogeochemical processes involved in cycling and decomposition of organic matter [80]. A total of 8 new rare actinomycete species were reported from seawater for the period mid-2013 to 2017 (Table 3), among which 2 novel genera, *Pontimonas* and *Tamlicoccus*. They belong to six actinomycete families: Nocardioidaceae (3 new species), Cellulomonadaceae (1 new species), Micrococcaceae (1 new species), Microbacteriaceae (1 new species), Dermacoccaceae (1 new species) and Dermabacteraceae (1 new species). From these studies, it is apparent that lower numbers of rare actinomycetes are isolated from seawater than from sediments. However, novel genera have been reported from seawater, which contribute to the extension of phylogenetic diversity of rare actinomycetes (Table 3) [7].

A substantial number of rare actinomycetes were reported to be associated to various members of marine benthic communities, such as sponges, corals, ascidians, sea anemones, sea cucumbers, sea urchins and seaweeds [7,62,81]. Five novel genera, 17 new rare actinomycete species belonging to 11 different actinomycete families were reported from marine plants and animals between 2007 and mid-2013 [7]. A total of 14 new species of rare actinomycetes belonging to 12 different families have been reported from various sponges, corals, algae, sea urchin, jelly fish and fish between mid-2013 and 2017 (Table 4). The families of novel (potentially symbiotic) actinomycete species reported from mid-2013 to 2017 are Micromonosporaceae (2 new species), Pseudonocardiaceae (2 new species), Microbacteriaceae (1 new species), Mycobacteriaceae (1 new species), Nocardioidaceae (1 new species), Micrococcaeae (1 new species), Intrasporangiaceae (1 new species), Nocardiaceae (1 new species), Rubrobacteraceae (1 new species), Actinosynnemataceae (1 new species), Gordoniaceae (1 new species) and Promicromonosporaceae (1 new species). Thus, marine organisms remain a rich source of novel rare actinomycetes (Table 4) and a substantial number of host-associated rare actinomycete genera have not been reported from other marine habitats (*Labedella*, *Phycicola*, *Iamia*, *Euzebya* and *Koreibacter*) [7]. Interestingly, *Microbacterium aureliae* was reported for the first time from *Aurelia aurita*, the moon jellyfish.

Mangrove forests are highly dynamic ecosystems that cover and protect approximately 75% of the world’s tropical and subtropical coastal areas [128] and harbor a rich diversity of marine, freshwater and terrestrial flora and fauna. The diversity of the microbial community in mangrove environments is still rather unexplored [60]. The large fluctuation of salinity and tidal gradients make the mangrove forests unique environments that favors the production of unusual metabolites among the residing microorganisms [60]. Novel actinomycetes reported from different mangrove habitats including sediments, mangrove plant rhizosphere soil and mangrove endophytes are classified into 25 genera, 11 families and 8 suborders [7,129]. A total of 27 new species of rare actinomycetes belonging to 13 different families have been reported from mangrove habitats for the period mid-2013–2017 (Table 5). Among them, two novel genera, *Mamia* and *Monashia*, were reported to be isolated from the 20 cm top-layer of mangrove soil. The families reported in mangrove sediments between mid-2013 and 2017 are Demequinaceae (9 new species), Micromonosporaceae (5 new species), Nocardiopsaceae (2 new species), Micrococcaceae (2 new species), Nocardioidaceae (1 new species), Intrasporangiaceae (1 new species), Pseudonocardiaceae (1 new species), Microbacteriaceae (1 new species), Thermomonosporaceae (1 new species), Jiangellaceae (1 new species), Beutenbergiaceae (1 new species), Streptosporangiaceae (1 new species) and Kineosporiaceae (1 new species). 

### 3.2. Marine Rare Actinomycetes Diversity: A Decade of Experience (2007–2017) 

**Figure 1:**
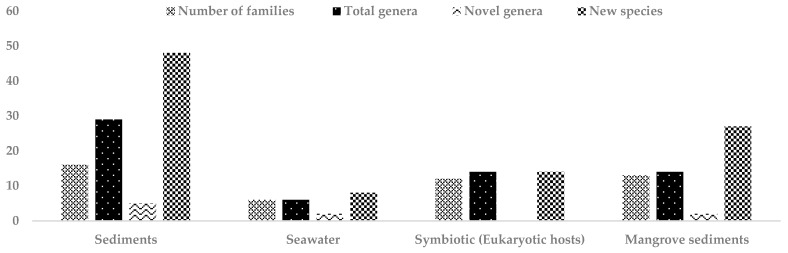
Total number of families, novel genera and new species of rare actinomycetes reported from different marine habitats between mid-2013 and 2017.

In summary, a total of 97 new species belonging to 27 different rare actinomycete genera, of which 9 represent novel genera, were reported, from the marine environment between mid-2013 and 2017 (Table 2, Table 3, Table 4, Table 5 and Table 6; Figure 1). Furthermore, the families Pseudonocardiaceae, Demequinaceae, Micromonosporaceae and Nocardioidaceae were most frequently isolated from the marine environment. For the period 2007-mid 2013, 80 new species belonging to 23 families of marine rare actinomycetes were reported (Table 6). These data show that the discovery rate of new rare actinomycetes from marine habitats is steady. Interestingly, isolates from 10 actinomycete families, such as Actinomycetaceae, Actinopolysporaceae, Brevibacteriaceae, Rubrobacteraceae, Actinosynnemataceae, Gordoniaceae, Jiangellaceae, Kineosporiaceae, Dermacoccaceae and Dermabacteraceae were reported for the period between mid-2013 and 2017 that were not reported for the period 2007 to mid-2013. Cumulatively this means that a total of of 177 new species of rare actinomycetes representing 33 families including 3 novel families and 29 novel genera were reported from marine habitats in the last 10 years (Table 6). Actinomycete families such as Micromonosporaceae, Nocardioidaceae, Pseudonocardiaceae, Microbacteriaceae, Micrococcaceae, Demequinaceae, Nocardiopsaceae, Propionibacteriaceae and Intrasporangiaceae are the families most frequently reported from marine habitats during this period. However, no novel actinomycete family has been reported from marine habitats since mid-2013.

## 4. Actinomycetes as Sources of Antibiotics 

Actinomycetes has been one of the most fertile sources for the discovery of new antibiotics since they were first discovered and a number of the antibiotics currently in use are natural products or analogs of natural products from actinomycetes [153]. Actinomycin was the first antibiotic discovered from actinomycetes in 1940 from a culture of *Streptomyces antibioticus* [154], followed by streptothricin from *Streptomyces lavendulae* in 1942 [155], and streptomycin from *Streptomyces griseus* in 1944 [156]. *Streptomyces* species have been the key source of clinical antibiotics, and more than 80% of all antibiotics of actinomycetes origin have been derived from this single genus [3,157]. Out of all microbially-derived antibiotic classes, 10 classes are exclusively produced by actinomycetes. Those are polyene macrolides, oligomycin-type large-membered macrolides, daunomycin-type anthracyclines, nigericin-type polyether antibiotics, nonactin-type cyclopolylactones, aminoglycosides, anthracyclines, streptothricins, actinomycins and quinoxaline-peptides [3]. The antibiotics production of different actinomycete strains can vary enormously as some actinomycete species produce a single antibiotic, whereas some produce a wide-range of different compounds and compound classes [5]. A total of 30 new antibiotics have been launched worldwide since 2000. Of the 30 new antibiotics, 2 were natural products (NP), 12 were NP-derived and 16 were synthetic antibiotics [158]. Out of these 30 new antibiotics, 12 were reported from members of actinomycetes, either as natural product or natural product-derivatives representing 7 different antibiotic classes (Table 7). Due to the decline in the number of new chemical scaffolds and rediscovery of known molecules, the innovation in antibiotic development has slowed down. The exploration of alternative taxa, which have not been previously cultivated, could alleviate urgent needs related to resistance against currently used antibiotics.

### 4.1. Rare Actinomycetes: A Target for Future Drugs

As a result, rare actinomycetes are becoming an increasingly important focus of investigation in the search for novel natural products because (1) they occupy a poorly explored taxonomic and environmental space, which reduces the likelihood of replication of discovery, and (2) the phylum Actinobacteria is a rich source of bioactive secondary metabolites [46] that can be expected to yield novel chemical scaffolds for the development of new antibiotics.

### 4.2. Marine Rare Actinomycetes Is a Source of Antibiotics

Approximately 100 new bioactive compounds were reported from 38 rare actinomycete strains belonging to 15 genera described between 2007 and mid-2013. Out of these 15 different genera, *Salinispora* (20 new compounds), *Verrucosispora* (18 new compounds), *Nocardiopsis* (12 new compounds), *Actinoalloteichus* (11 new compounds), *Marinispora* (10 new compounds) and *Micromonospora* (9 new compounds) were predominant for discovery of novel secondary metabolites from 2007 to mid-2013 [7]. A total of 4 compounds derived from marine actinomycetes are currently in clinical trials (Table 7) of which 3 were obtained from marine *Salinispora* spp. indicating that *Streptomyces* spp. are no longer the most important biological resource for new antibiotics.

### 4.3. Novel/New Compounds from Marine Rare Actinomycetes between mid-2013 and 2017

**Figure 2:**
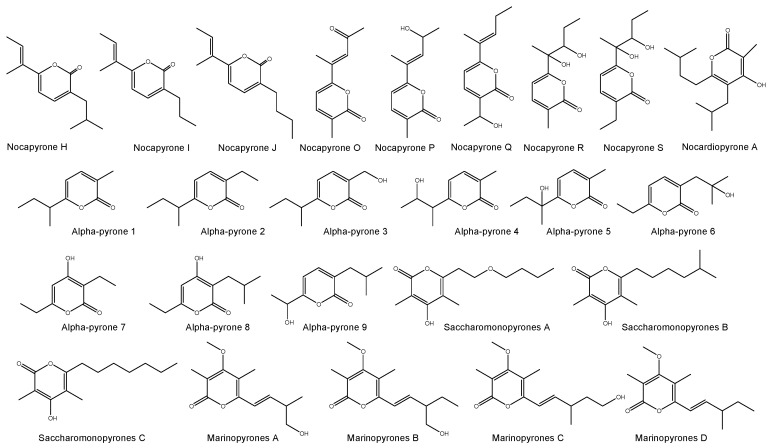
New pyrones produced by marine rare *Nocardiopsis* spp., *Streptomonospora* sp. and *Saccharomonospora* sp. between mid-2013 and 2017.

**Figure 3:**
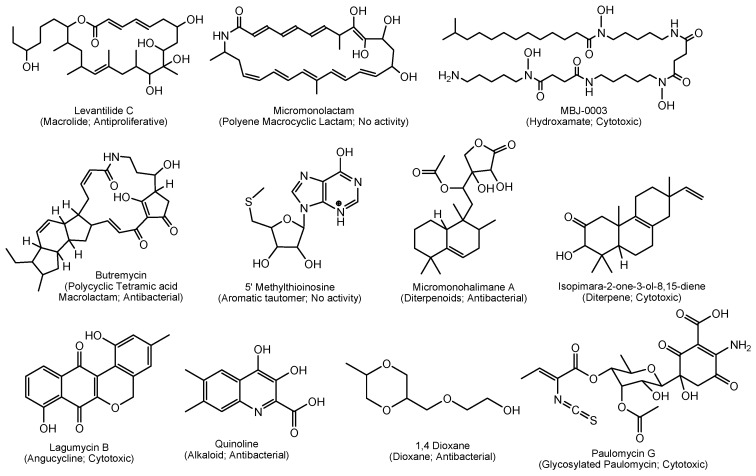
Some novel natural products from marine *Micromonospora* spp. reported from mid-2013 to 2017.

**Figure 4:**
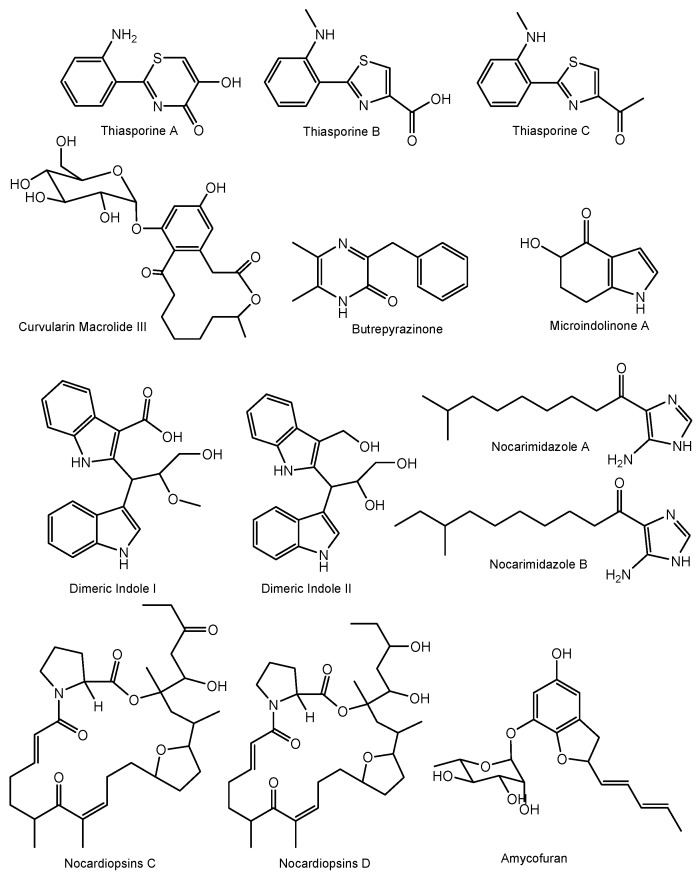
Some of the unique chemical moieties in natural products produced by marine rare actinomycetes between mid-2013 and 2017.

**Figure 5:**
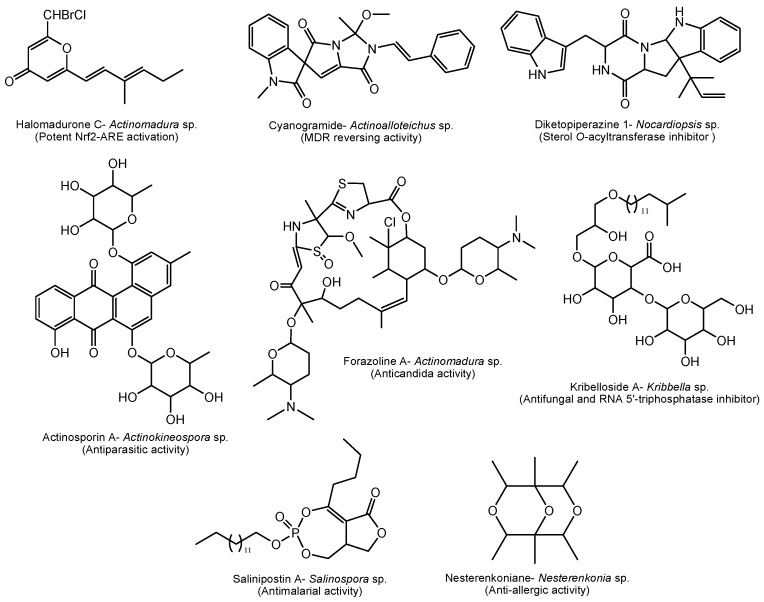
Some unusual biologically active compounds produced by marine rare actinomycetes between mid-2013 and 2017.

A total of 167 different new bioactive compounds were reported from 58 rare actinomycete strains belonging to 24 genera from mid-2013 to 2017 (Table 8). Among them, genera such as *Nocardiopsis* (40 new compounds), *Micromonospora* (37 new compounds), *Salinispora* (21 new compounds) and *Pseudonocardia* (14 new compounds) are leading with respect to the number of novel secondary metabolites (Table 8). Among them, there are new/novel pyrones, structurally diverse natural products and unique chemical moieties (Figure 2, Figure 3, Figure 4 and Figure 5). 

A total of 7 different chemical classes of natural products were reported from marine *Nocardiopsis* spp. between mid-2013 and 2017 of which, α-pyrones (18 out of 40 compounds) were predominant (Table 9; Figure 2). These molecules have a wide range of biological activities, such as *pro*-inflammatory activity (enhancing and stimulating the inflammatory response), anti-inflammatory activity, antibacterial and cytotoxic activities (Table 9). In addition, the genera *Streptomonospora* and *Saccharomonospora* also produce a substantial number of α-pyrones. Besides, nocarimidazoles from *Nocardiopsis* sp. possess a 4-aminoimidazole ring rarely found in microbial secondary metabolites [162] and rare prolinyl-macrolactam polyketides were isolated from *Nocardiopsis* sp. [163]. Sterol *O*-acyltransferase [SOAT, also known as acyl-CoA: cholesterol acyltransferase (ACAT)], an endoplasmic reticulum membrane protein, catalyzes the synthesis of cholesteryl ester from free cholesterol and long-chain fatty acyl-CoA. SOAT has been postulated as a target for modulation by a new type of antiatherosclerotic agent. Interestingly, a diketopiperazine derived from marine *Nocardiopsis* sp. was found to be an effective SOAT inhibitor [164].

Marine *Micromonospora* spp. produced 37 out of the 167 compounds reported from mid-2013–2017 and were chemically diverse (Table 9; Figure 3). In total, 13 chemical classes, including macrolides, polyene macrocyclic lactams, polycyclic tetramic acid macrolactams, aromatic tautomers, hydroxamates, diterpenoids, diterpenes, angucyclines, quinolone alkaloids, dioxanes, glycosylated paulomycins, glycosides and aglycone spirotetrorates were identified in *Micromonospora* spp. during this period. Polyene macrolactams are an underexplored group of natural products that have only been found in actinomycetes. Micromonolactam is a new polyene macrocyclic lactam isolated from a marine *Micromonospora* sp. (Figure 3). However, micromonolactam did not show antibacterial activities against test pathogens [165]. Another interesting group of natural products, paulomycins, are glycosylated molecules containing a pauloate residue that are of pharmacological interest due to their strong antibiotic properties [166]. Paulomycin G is structurally unique because it is the smallest bioactive paulomycin in the paulomycin family of antibiotics, lacking the paulomycose moiety (Figure 3). Furthermore, a number of novel chemical skeletal structures are reported from marine *Micromonospora* spp. For example, polycyclic tetramic acid macrolactams of butremycin [167], halimane-type diterpenoids of micromonohalimanes [168] and a novel pimarane diterpene in isopimara-2-one-3-ol-8,15-diene [169] (Figure 3).

Additionally, other rare actinomycete genera have yielded a number of unique chemical moieties, which were not previously reported from microbially-derived natural products (Figure 4). For example, thiasporine A is the first natural product with a 5- hydroxy-4H-1,3-thiazin-4-one moiety, along with two new thiazole derivatives and were reported from *Actinomycetospora chlora* [170] (Figure 4). Other unusual structures include a curvularin macrolide with a rare α-D-glucopyranose substituent from *Pseudonocardia* sp. [171]; a butrepyrazinone, from *Verrucosispora* sp. that possesses an unusual methylation pattern on the pyrazinone ring [172], a novel indole microindolinone A from *Microbacterium* sp. [173], new dimeric indole derivatives with acetylcholinesterase (AchE) inhibitory activity from *Rubrobacter radiotolerans* [174] and a structurally new amycofuran bearing a rhamnose sugar from *Amycolatopsis* sp. [175]. 

*Actinomadura* sp. derived halomadurones (Figure 5) demonstrated potent nuclear factor E2-related factor antioxidant response element (Nrf2-ARE) activation, which is an important therapeutic approach for treatment of neurodegenerative diseases [176]. Cyanogramide obtained from *Actinoalloteichus cyanogriseus* showed efficient anticancer activity by reversing the adriamycin-induced resistance of K562/A02 and MCF-7/Adr cells, and the vincristine-induced resistance of KB/VCR cells [177].

A marine sponge-derived *Actinokineospora* sp. produces actinosporins with selective activity against the parasite *Trypanosoma brucei brucei*, the causative agent of sleeping sickness [182]. Fungal infections, particularly candidiasis, is one of the serious diseases worldwide. A novel antifungal polyketide, forazoline A isolated from *Actinomadura* sp. showing significant activity against *Candida albicans* works through a new mechanism of action by disrupting membrane integrity [186]. Another way of controlling candidiasis is by capping enzyme repressors. Inhibitors of the enzyme RNA 5’-triphosphatase in yeast may be used against pathogenic yeasts such as *Candida*. Interestingly, novel kribellosides from a marine *Kribbella* sp. inhibit activity of Cet1p (RNA 5’-triphosphatase) from *Saccharomyces cerevisiae in vitro* [208]. Another interesting biological activity is anti-allergic activity shown by nesterenkoniane, a novel cyclic ether isolated from the deep-sea-derived *Nesterenkonia flava*. Nesterenkoniane is the first report on secondary metabolites from the genus of *Nesterenkonia* [202]. Furthermore, discovery of anti-malarial drugs is one of the targets of research in pharma industries. Salinipostin A, isolated from the marine genus *Salinispora* shows potent antimalarial activity against *Plasmodium falciparum* growth (EC_50_ = 50 nM) and a high selectivity index (SI > 10^3^) [192] (Figure 5).

### 4.4. Genome Mining of Marine Rare Actinomycetes 

The rapid development of genome and metagenome sequencing methods including identification of secondary metabolite gene clusters has lead to the discovery of the genetic machinery encoding for novel natural products from microorganisms that have not yet been chemically identified [211]. The majority of these gene clusters encode for polyketides (PK), non-ribosomally synthesized peptides (NRP), ribosomally and post-translationally modified peptides (RiPPs) and aminoglycosides [211]. The bioinformatic analysis of genomes can also reveal silent secondary metabolite gene clusters, which are not expressed under standard laboratory conditions [212]. More than 23,000 PK and NRP have been reported so far, many of them found in actinomycetes, and they are being widely tested for pharmaceutical applications [213,214]. This approach has also been used for the identification of new antibiotic scaffolds from rare genera of actinomycetes from marine sediments [16]. Recently, Schorn and colleagues [215] have shown that rare marine actinomycetes-derived genomes demonstrated a high degree of novelty and diversity, with *Corynebacterium*, *Gordonia*, *Nocardiopsis*, *Saccharomonospora* and *Pseudonocardia* as genera representing the highest biosynthetic gene cluster diversity. A total of 13 new bioactive compounds have been derived from marine rare actinomycetes, such as *Saccharomonospora* sp., *Salinispora* spp., *Micromonospora* spp. and *Streptosporangium* sp. using genome-based approaches between mid 2013 and 2017 (Table 10). 

These numbers of new biosynthetic gene clusters and corresponding compounds will undoubtly increase in the near future due to revolutionary developments in the genome- and metagenome-based approaches for drug discovery [215] and it likely that omics-based screening for novel bioactive compounds will prevail over microbial isolation as the most efficient method for first identification of bioactivity potential of strains and environmental samples [216].

## 5. Conclusions

In the last decade (2007–2017), a great range of diverse, new and rare actinomycetes have been isolated from the marine environment. Employment of heat-treatment of marine sediment samples, the use of low-nutrient agar medium (seawater agar) or a growth medium with natural seawater along with the use of antifungal agents, favor the isolation of marine rare actinomycetes. At least 177 new species, which represent 29 novel genera and 3 novel families, were obtained as pure cultures. Micromonosporaceae, Nocardioidaceae, Pseudonocardiaceae, Microbacteriaceae, Micrococcaceae, Demequinaceae, Nocardiopsaceae, Propionibacteriaceae and Intrasporangiaceae were the families most frequently isolated from the marine environment. 

In total, 267 new natural products derived from 96 different marine rare actinomycete strains belonging to 28 genera have been reported from 2007 to 2017. Out of these 28 marine genera, *Nocardiopsis*, *Micromonospora*, *Salinispora*, *Verrucosispora*, *Pseudonocardia* and *Actinoalloteichus* are topmost producers of novel new secondary metabolites.

The rare actinomycetes isolated and biomolecules discovered represent most likely only the low-hanging fruits and the immense diversity of microorganisms in marine habitats as shown from large cultivation-independent studies [225,226] are the proof for the presence of an even larger diversity of currently uncultivable rare actinomycetes and putative secondary metabolites. This uncultured majority should be the target of future selective isolation strategies and procedures. In addition, genetic engineering of whole biosynthetic gene clusters is finally gaining ground [216] and may be the key to access hidden gene clusters from rare actinomycetes. A breakthrough in heterologous expression would herald ‘another golden age’ of novel bioactive natural product discovery, for which marine rare actinomycetes may be one of the important sources.

## Figures and Tables

**Table 1:** Pre-treatment methods for the selective isolation of marine rare actinomycetes.

Pre-treatment	Marine Source	Isolation Medium	Incubation Temperature/Time	Target Rare Genera	Ref.
***Heat***					
Incubation in water bath at 50 °C for 60 min	WS	Starch-casein agar + 10 µg nalidixic acid, 25 µg nystatin and 10 µg cycloheximide	24 °C for 28 days	*Micromonospora*	[45]
	WS	M1 agar + 75 µg cycloheximide	20–24 °C for 14–28 days	*Micromonospora*	[49]
50 °C for 15 min	WS	Starch-casein agar + 10 µg nystatin and 25 µg cycloheximide	28 °C for 7 days	*Monashia, Microbacterium* and *Sinomonas*	[23,50,51]
55 °C for 20 min	WS	Glucose peptone tryptone agar + 50 mg nystatin, 50 mg cycloheximide, 25 mg novobiocin and 20 mg nalidixic acid	28 °C for 21 days	*Micromonospora*	[52]
Incubation in water bath at 55 °C for 6 min	DS	M1–M5 agar + 100 µg cycloheximide and 5 µg rifampin	25–28 °C for 2–6 weeks	*Micromonospora* and *Salinispora*	[11,53]
Incubation in water bath at 60 °C for 10 min	DS	M1–M12 agar + 100 µg cycloheximide and 50 µg nystatin	28 °C for 3 months	*Micromonospora* and *Salinispora*	[28]
Speedvac 30 °C, 16 h; 120 °C, 60 min	DS	Different selective media + cycloheximide (50 μg/mL), nystatin (75 μg/mL) and nalidixic acid (30 μg/mL)	20 °C for 2–6 weeks	Rare actinomycetes	[54]
41 °C for 10, 30 and 60 days	DS	Different selective media	28 °C for 2–3 weeks	*Streptoverticillium, Catellatospora, Nocardia* and *Actinopolyspora*	[55]
70 °C for 15 min	WS	Different selective media	25 °C for 4 weeks	*Micromonospora, Microbispora, Actinoplanes* and *Actinomadura*	[56]
55 °C for 15 min	DS	Asparagine-glucose agar medium + nalidixic acid (25 μg/mL) and secnidazole (25 μg/mL)	25 °C for 2 weeks	*Pseudonocardia*	[57]
45, 55 or 65 °C for 30 min	WS	ISP-3 and ISP-4 + cycloheximide (50 μg/mL), nystatin (50 μg/mL), and nalidixic acid (20 μg/mL)	27 °C for 3 weeks	*Micromonospora*	[58]
60 °C for 6 min	WS	M1 medium and Glucose-yeast extract medium + nystatin (50 μg/mL), and nalidixic acid (10 μg/mL)	25 °C for 6 weeks	*Nocardia, Nonomuraea, Rhodococcus, Saccharopolyspora* and *Gordonia*	[59]
***Physical***					
Dry in laminar air flow hood; Stamping	WS/DS	M1–M12 agar + 100 µg cycloheximide and 50 µg nystatin	28 °C for 3 months	*Micromonospora* and *Salinispora*	[28,53]
***Mechanic***					
Shake with glass beads for 30 s and settled for 5 min	WS	Different selective media + cycloheximide (50 μg/mL), nystatin (75 μg/mL) and nalidixic acid (30 μg/mL)	20 °C for 6 weeks	Rare actinomycetes	[54]
***Chemical/+ Heat***					
1.5% phenol + 30 min at 30 °C	DS	Different selective media + cycloheximide (50 μg/mL), nystatin (75 μg/mL) and nalidixic acid (30 μg/mL)	20 °C for 2–6 weeks	*Micromonospora*	[4]
0.02% benzethonium chloride + 30 min at 30 °C	DS	Different selective media + cycloheximide (50 μg/mL), nystatin (75 μg/mL) and nalidixic acid (30 μg/mL)	20 °C for 2–6 weeks	Rare actinomycetes	[54]
0.05% SDS and 6% yeast extract (40 °C, 200 rpm, 30 min)	DS	Different selective media + cycloheximide (25–100 μg/mL) and nystatin (25–50 μg/mL)	28 °C for 1–12 weeks	*Actinomadura, Micromonospora, Nocardia, Nonomuraea, Rhodococcus* and *Verrucosispora*	[60]
1.5% phenol	DS	Different selective media + cycloheximide (50 μg/mL), nystatin (75 μg/mL) and nalidixic acid (30 μg/mL)	20 °C for 2–6 weeks	Rare actinomycetes	[54]
Chloramine-T	DS	Different selective media + cycloheximide (25–100 μg/mL) and nystatin (25–50 μg/mL)	28 °C for 1–12 weeks	*Actinomadura, Micromonospora, Nocardia, Nonomuraea, Rhodococcus, Streptomyces* and *Verrucosispora*	[60]
***Centrifugation***					
Differential centrifugation	WS	Selective media	28 °C for 12 weeks	*Micromonospora, Rhodococcus* and *Streptomyces*	[30]
***Freezing***					
Freeze (−20 °C, 24 h), thawed, dilution	WS	M1-M12 agar + 100 µg cycloheximide and 50 µg nystatin	28 °C for 3 months	*Micromonospora* and *Salinispora*	[28]
Freeze at −18 °C	WS	Different selective media + nystatin (50 μg/mL) and nalidixic acid (10 μg/mL)	28 °C for 2–3 weeks	*Nocardiopsis, Nocardia* and *Streptosporangium*	[61]
***Radiation***					
UV irradiation for 30 s (distance 20 cm, 254 nm, 15 W)	WS	Different selective media + nystatin (50 μg/mL) and nalidixic acid (10 μg/mL)	28 °C for 2–3 weeks	*Nocardiopsis, Nocardia* and *Pseudonocardia*	[61]
Superhigh frequency radiation inmicrowave oven for 45 s (2460 MHz, 80 W)	WS	Different selective media + nystatin (50 μg/mL) and nalidixic acid (10 μg/mL)	28 °C for 2–3 weeks	*Streptosporangium* and *Rhodococcus*	[61]
Extremely high frequency radiation (1 kHz within wavelength band of 8–11.5 mm)	WS	Different selective media + nystatin (50 μg/mL) and nalidixic acid (10 μg/mL)	28 °C for 2–3 weeks	*Nocardiopsis, Nocardia* and *Streptosporangium*	[61]

WS: Wet Sediment; DS: Dried Sediment.

**Table 2:** New species of rare actinomycetes from marine sediments reported during the period of mid 2013–2017.

Strain/Family	Nature of Sample	Isolation Medium	Ref.
*Saccharomonospora amisosensis*/Pseudonocardiaceae	Deep marine sediment at a depth of 60 m	SM3 medium (yeast nitrogen base 67.0 g, casamino acids 100 mg were added to a litre of distilled water and the solution sterilised using cellulose filters (0.20 mm) prior to the addition of sterilised di-potassium hydrogen phosphate (200 mL; 10%, w/v); 100 mL of this basal medium was added to 900 mL of sterilised molten agar (1.5%, w/v) followed by filter sterilised solutions of D(+) melezitose (1%, w/v), cycloheximide (50 µg mL^−1^), neomycin sulphate (4 µg mL^−1^) and nystatin (50 µg mL^−1^)	[82]
*Saccharomonospora oceani*/Pseudonocardiaceae	Marine sediment	Trypticase soy broth agar (DSMZ Medium 535)	[83]
*Actinophytocola sediminis*/Pseudonocardiaceae	Marine sediment at a depth of 2439 m	Starch casein nitrate agar medium (10.0 g soluble starch, 0.3 g casein, 2 g KNO_3_, 0.05 g MgSO_4_.7H_2_O, 35 g NaCl, 2 g K_2_HPO_4_, 0.02 g CaCO_3_, 10 mg FeSO_4_, 20 g agar, distilled water 1 L)	[84]
*Pseudonocardia sediminis*/Pseudonocardiaceae	Sea sediment at a depth of 652 m	DSMZ 621 medium (250 mg each of Bacto peptone (Difco), Bacto yeast extract and glucose, as well as 20 mL Hutner’s basal salts medium, 10 mL vitamin solution no. 6, 35 g NaCl and 1000 mL distilled water)	[85]
*Amycolatopsis flava*/Pseudonocardiaceae	Marine sediment	CMKA medium [(L^−1^) 0.5 g casein hydrolysate, 1.5 g mannitol, 1 g KNO_3_, 2 g (NH_4_)_2_SO_4_, 0.5 g K_2_HPO_4_, 0.5 g CaCO_3_, 20 g agar]. The multi-salts comprised of 49% (w/w) MgCl_2_.6H_2_O, 32% (w/w) NaCl, 14 % (w/w) CaCl_2_ and 5 % (w/w) KCl	[86]
*Saccharopolyspora griseoalba*/Pseudonocardiaceae	Marine sediment	CMKA medium [(L^−1^) 0.5 g casein hydrolysate, 1.5 g mannitol, 1 g KNO_3_, 2 g (NH_4_)_2_SO_4_, 0.5 g K_2_HPO_4_, 0.5 g CaCO_3_, 20 g agar]. The multi-salts comprised of 49% (w/w) MgCl_2_.6H_2_O, 32% (w/w) NaCl, 14 % (w/w) CaCl_2_ and 5 % (w/w) KCl	[87]
*Amycolatopsis albispora*/Pseudonocardiaceae	Deep-sea sediment at a depth of −2945 m	Modified Zobell 2216E agar (1.0 g yeast extract, 5.0 g tryptone, 34 g NaCl, 15 g agar and 1 L distilled water)	[88]
*Pseudonocardia profundimaris*/Pseudonocardiaceae	Marine sediment at a depth of −7118 m	Modified ZoBell 2216E agar plates (0.5% tryptone, 0.1% yeast extract, 3.4% sodium chloride and 1.8% agar)	[89]
*Nocardioides pacificus*/Nocardioidaceae	Deep sub-seafloor sediment at a depth of 107.3–107.4 m	Marine agar 2216 (Difco)	[90]
*Nocardioides nanhaiensis*/Nocardioidaceae	Sea sediment at a depth of 880 m	DSMZ 621 medium (250 mg each of Bacto peptone (Difco), Bacto yeast extract and glucose, as well as 20 mL Hutner’s basal salts medium, 10 mL vitamin solution no. 6, 35 g NaCl and 1000 mL distilled water)	[91]
*Nocardioides antarcticus*/Nocardioidaceae	Marine sediment	Marine agar 2216 (Becton Dickinson)	[92]
*Nocardioides litoris*/Nocardioidaceae	Marine beach sediment	Starch casein agar (1% soluble starch, 0.03% casein, 0.2% KNO_3_, 0.2% NaCl, 0.005% MgSO_4_.7H_2_O, 0.2% K_2_HPO_4_, 0.02% CaCO_3_, 0.001% FeSO_4_.7H_2_O, 1.8% agar)	[93]
*Nocardioides flavus*/Nocardioidaceae	Marine sediment at a depth of −7068 m	Seawater agar (15.0 g agar and 1 L natural seawater)	[35]
*Streptomonospora sediminis*/Nocardiopsaceae	Marine sediment	Agar medium (glycerine 10.0 g, l-arginine 5.0 g, (NH_4_)_2_SO_4_ 2.64 g; KH_2_PO_4_ 2.38 g, K_2_HPO_4_ 5.65 g, MgSO_4_.7H_2_O 1.0 g, CuSO_4_.5H_2_O 0.0064 g, FeSO_4_.7H_2_O 0.0011 g; MnCl_2_.4H_2_O 0.0079 g; ZnSO_4_.7H_2_O 0.0015 g, agar 15.0 g; distilled water 1.0 L)	[94]
*Streptomonospora nanhaiensis*/Nocardiopsaceae	Marine sediment at a depth of 2918 m	Agar medium (glycerine 10.0 g, l-arginine 5.0 g, (NH_4_)_2_SO_4_ 2.64 g; KH_2_PO_4_ 2.38 g, K_2_HPO_4_ 5.65 g, MgSO_4_.7H_2_O 1.0 g, CuSO_4_.5H_2_O 0.0064 g, FeSO_4_.7H_2_O 0.0011 g; MnCl_2_.4H_2_O 0.0079 g; ZnSO_4_.7H_2_O 0.0015 g, agar 15.0 g; distilled water 1.0 L)	[94]
*Nocardiopsis oceani*/Nocardiopsaceae	Marine sediment at a depth of 2460 m	Gauze’s synthetic medium no. 1 (soluble starch 20.0 g, KNO_3_ 1.0 g, NaCl 0.5 g, MgSO_4_.7H_2_O, 0.5 g, K_2_HPO_4_ 0.5 g, FeSO_4_.7H_2_O 10.0 mg, agar 15.0 g and distilled water 1.0 L)	[95]
*Nocardiopsis nanhaiensis*/Nocardiopsaceae			
*Microbacterium hydrothermale*/Microbacteriaceae	Hydrothermal sediment at a depth of 2943 m	Modified ZoBell 2216E agar plates (0.5% tryptone, 0.1% yeast extract, 3.4% sodium chloride and 1.8% agar)	[96]
*Agromyces marinus*/Microbacteriaceae	Sea sediment	NBRC medium 802 [Polypepton (Wako) 2 g, yeast extract 0.4 g, MgSO_4_.7H_2_O 0.2 g and agar 15 g in 1.0 L distilled water supplemented with NaCl (30 g^−l^), cycloheximide (50 mg^−l^) and nalidixic acid (20 mg^−l^)].	[97]
*Microbacterium enclense*/Microbacteriaceae	Marine sediment	Marine agar (HiMedia)	[98]
*Microbacterium nanhaiense*/Microbacteriaceae	Sea sediment at a depth of 2093 m	Yeast extract/malt extract agar (1 L seawater, 0.5 g malt extract, 0.2 g yeast extract, 0.1 g glucose and 20 g agar)	[99]
*Zhihengliuella flava*/Micrococcaceae	Sea sediment	NBRC medium 802 (0.2% polypeptone, 0.04% yeast extract, 0.02% MgSO_4_.7H_2_O and 1.5% agar)	[100]
*Kocuria indica*/Micrococcaceae	Marine sediment	Marine agar 2216 (Difco)	[101]
*Nesterenkonia alkaliphila*/Micrococcaceae	Deep-sea sediment at a depth of 7118 m	Modified ISP 1 (1 L natural seawater, 10 g glucose, 5 g peptone, 5 g yeast extract, 0.2 g MgSO_4_.7H_2_O, 10 g NaHCO_3_, 27 g Na_2_CO_3_.10H_2_O and 15 g agar)	[102]
*Kocuria subflava*/Micrococcaceae	Marine sediment	No. 38 medium [(L^−1^) yeast extract 0.4 g; glucose 0.4 g; malt extract 0.4 g; B-vitamin trace 1 mL (0.5 mg each of thiamine-HCl (B1), riboflavin, niacin, pyridoxin, ca-pantothenate, inositol, *p*-aminobenzoic acid, and 0.25 mg of biotin, agar 15 g, distilled water 1000 mL]	[103]
*Luteococcus sediminum*/Propionibacteriaceae	Deep subseafloor sediment	Marine agar 2216 (Difco)	[104]
*Mariniluteicoccus flavus* (novel genus)/Propionibacteriaceae	Deep-sea sediment at a depth of 2439 m	HP agar medium (5 g fucose, 1 g proline, 1 g (NH_4_)_2_SO_4_, 2 g CaCl_2_, 1 g K_2_HPO_4_, B vitamin mixture (0.5 mg each thiamine hydrochloride, riboflavin, niacin, pyridoxine, calcium pantothenate, inositol and *p*-aminobenzoic acid and 0.25 mg biotin), 35 g NaCl, 12 g agar, 1000 mL distilled water)	[43]
*Tessaracoccus lapidicaptus*/Propionibacteriaceae	Deep subsurface sediment at a depth of 297 m	Anoxic F4 medium (0.4 g NaCl, 0.4 g NH_4_Cl, 0.3 g MgCl_2_.6H_2_O, 0.05 g CaCl_2_.2H_2_O, 1 g yeast extract, 2 g peptone, 1 g glucose, 1 g succinic anhydride, 7.5 g NaHCO_3_, 0.5 g KH_2_PO_4_, 0.5 g Na_2_S, 1 mg resazurin and 1 L distilled water)	[105]
*Tessaracoccus arenae*/Propionibacteriaceae	Sea sediment	Marine agar 2216 (Difco)	[106]
*Rhodococcus enclensis*/Nocardiaceae	Marine sediment	Marine agar 2216 (Difco)	[107]
*Nocardia jiangsuensis*/Nocardiaceae	Coastal sediment	Starch arginine agar (2.5 g soluble starch, 1.0 g arginine, 1.0 g (NH_4_)_2_SO_4_, 2.0 g CaCl_2_, 1.0 g K_2_HPO_4_, 0.2 g MgSO_4_.7H_2_O, 10 mg FeSO_4_.7H_2_O, 15.0 g agar supplemented with 3% (w/v) NaCl, nystatin and nalidixic acid)	[108]
*Micromonospora fluostatini*/Micromonosporaceae	Marine sediment	M1 medium (10 g soluble starch, 4 g yeast extract, 2 g peptone, 18 g agar, and 1 L of natural seawater)	[109]
*Micromonospora yasonensis*/Micromonosporaceae	Marine sediment at a depth of 45 m	SM3 medium (Gauze’s medium 2) [20 g casaminoacids, 20 g soluble starch, 4 g yeast extract, 15 g agar, 1 L distilled water] supplemented with filter sterilised cycloheximide (50 µg mL^−1^), nalidixic acid (10 µg mL^−1^), novobiocin (10 µg mL^−1^) and nystatin (50 µg mL^−1^)	[110]
*Micromonospora profundi*/Micromonosporaceae	Marine sediment at a depth of 45 m	ISP 2 medium (yeast extract 4.0 g, malt extract 10.0 g, dextrose 4.0 g, distilled water 1 L and Bacto agar 20.0 g)	[111]
*Demequina activiva*/Demequinaceae	Tidal flat sediment	Marine agar 2216 (Becton Dickinson)	[112]
*Demequina litorisediminis*/Demequinaceae	Tidal flat sediment	Marine agar 2216 (Difco)	[113]
*Janibacter cremeus*/Intrasporangiaceae	Sea sediment	NBRC medium 802 (1.0% polypeptone, 0.2% yeast extract, 0.1% MgSO4.7H_2_O and 1.5% agar)	[114]
*Janibacter indicus*/Intrasporangiaceae	Hydrothermal sediment	ZoBell 2216E agar (0.5% tryptone, 0.1% yeast extract, 3.4%sodium chloride and 1.8% agar)	[115]
*Georgenia sediminis*/Bogoriellaceae	Marine Sediment at a depth of 141 m	Marine agar 2216 (Becton Dickinson)	[116]
*Georgenia subflava*/Bogoriellaceae	Deep sea sediment at a depth of 6310 m water depth	Modified ZoBell 2216E agar (1.0 g yeast extract, 5.0 g tryptone, 1 L of clarified seawater, 15.0 g agar)	[117]
*Ilumatobacter nonamiense*/Acidimicrobiaceae	Seashore sediment	Medium R (NaCl 25 g, MgSO_4_.7H_2_O 9 g, CaCl_2_.2H_2_O 0.14 g, KCl 0.7 g, Na_2_.HPO_4_.12H_2_O 0.25 g, Na_2_-EDTA 30 mg, H_2_BO_3_ 34 mg, FeSO_4_.7H_2_O 10 mg, FeCl_3_.6H_2_O 1.452 mg, MnCl_2_.4H_2_O 4.32 mg, ZnCl_2_ 0.312 mg, CoCl_2_.6H_2_O 0.12 mg, NaBr 6.4 mg, Na_2_MoO.2H_2_O 0.63 mg, SrCl_2_.6H_2_O 3.04 mg, RbCl 0.1415 mg, LiCl 0.61 mg, KI 0.00655 mg, V_2_O_5_ 0.001785 mg, Cycloheximide 50 mg, Griseofulvin 25 mg, Nalidixic acid 20 mg, Aztreonam 40 mg, RPMI1640 500 mg, Eagle Medium 500 mg, l-Glutamine 15 mg, NaHCO_3_ 100 mg, Agar 20 g and Distilled water 1 L)	[118]
*Ilumatobacter coccineum*/Acidimicrobiaceae	Seashore sand		
*Sediminivirga luteola* (novel genus)/Brevibacteriaceae	Marine sediment at a depth of −5233 m	Isolation medium (10 g glucose, 5 g peptone, 5 g yeast extract, 0.2 g MgSO_4_.7H_2_O, 10 g NaHCO_3_, 27 g Na_2_CO_3_.10H_2_O, 20 g agar and 1 L natural seawater)	[25]
*Brevibacterium sediminis*/Brevibacteriaceae	Deep-sea sediment at a depth of −2461 m	ISP 2 medium (yeast extract 4.0 g, malt extract 10.0 g, dextrose 4.0 g, distilled water 1 L and Bacto agar 20.0 g)	[119]
*Halopolyspora alba* (novel genus)/Actinopolysporaceae	Sea sediment	CMKA medium [(0.5 g casein acids hydrolysate, 1.5 g mannitol, 1 g KNO_3_, 2 g (NH_4_)_2_SO_4_, 0.5 g K_2_HPO_4_, 0.5 g CaCO_3_, 20 g agar and 20% (w/v) multi-salts]. The multi-salts comprised 49% (w/w) MgCl_2_, 32% (w/w) NaCl, 14% (w/w) CaCl_2_ and 5% (w/w) KCl	[24]
*Haloactinomyces albus* (novel genus)/Actinopolysporaceae	Marine sediment	CMKA medium [(0.5 g casein acids hydrolysate, 1.5 g mannitol, 1 g KNO_3_, 2 g (NH_4_)_2_SO_4_, 0.5 g K_2_HPO_4_, 0.5 g CaCO_3_, 20 g agar and 20% (w/v) multi-salts]. The multi-salts comprised 49% (w/w) MgCl_2_, 32% (w/w) NaCl, 14% (w/w) CaCl_2_ and 5% (w/w) KCl	[26]
*Flaviflexus huanghaiensis* (novel genus)/Actinomycetaceae	Coastal sediment	Marine agar 2216 (Difco)	[120]
*Paraoerskovia sediminicola*/Cellulomonadaceae	Sea sediment	NBRC medium 802 (1.0% polypeptone, 0.2% yeast extract, 0.1% MgSO_4_.7H_2_O and 1.5% agar)	[121]

**Table 3:** New species of rare actinomycetes from seawater reported during the period of mid 2013–2017.

Strain/Family	Nature of Sample	Isolation Medium	Ref.
*Nocardioides marinquilinus*/Nocardioidaceae	Coastal seawater	Marine agar (Difco)	[122]
*Nocardioides salsibiostraticola*/Nocardioidaceae	Seawater at a depth of 1 m	R2A agar (Difco)	[123]
*Nocardioides rotundus*/Nocardioidaceae	Seawater at a depth of −7001 m	Modified ZoBell 2216E agar (1.0 g yeast extract, 5.0 g tryptone, 1 L clarificated seawater and 15.0 g agar)	[124]
*Cellulomonas marina*/Cellulomonadaceae	Deep-seawater at a depth of 2800 m	ISP 2 medium (yeast extract 4.0 g, malt extract 10.0 g, dextrose 4.0 g, distilled water 1 L and Bacto agar 20.0 g)	[125]
*Kocuria oceani*/Micrococcaceae	Deep-sea hydrothermal plume water at a depth of 2800 m	ISP 2 medium (yeast extract 4.0 g, malt extract 10.0 g, dextrose 4.0 g, distilled water 1 L and Bacto agar 20.0 g) and SMPS (0.1 g peptone, 0.5 g mannitol, 3 g sea salt, 1000 mL distilled water, pH 7.5) agar, supplemented with nalidixic acid, cycloheximide and nystatin (each at 25 μg mL^−1^).	[126]
*Pontimonas salivibrio* (novel genus)/Microbacteriaceae	Seawater	Marine agar (Difco)	[36]
*Tamlicoccus marinus* (novel genus)/Dermacoccaceae	Seawater	SC-SW agar (1% soluble starch, 0.03% casein, 0.2% KNO_3_, 0.2% NaCl, 0.2% KH_2_PO4, 0.002% CaCO_3_, 0.005% MgSO_4_.7H_2_O, 0.001% FeSO_4_.7H_2_O, 1.8% agar, 60% natural seawater and 40% distilled water)	[37]
*Brachybacterium aquaticum*/Dermabacteraceae	Seawater	Tryptic soy agar medium (HiMedia)	[127]

**Table 4:** New species of symbiotic rare actinomycetes from eukaryotic hosts reported during the period of mid 2013–2017.

Strain/Family	Nature of Sample	Isolation Medium	Reference
*Verrucosispora andamanensis*/Micromonosporaceae	Marine sponge *Xestospongia* sp.	Starch-casein nitrate seawater agar (10 g soluble starch, 1 g sodium caseinate, 0.5 g KH_2_PO_4_, 0.5 g MgSO_4_ and 18 g agar in 1 L of seawater)	[130]
*Micromonospora spongicola*/Micromonosporaceae	Marine sponge at a depth of 5 m	Starch-casein nitrate agar (10 g soluble starch, 1 g sodium caseinate, 2 g KNO_3_, 0.5 g KH_2_PO_4_, 0.5 g MgSO_4_ and 18 g agar in 1 L seawater)	[131]
*Prauserella coralliicola*/Pseudonocardiaceae	Marine coral *Galaxea fascicularis* at a depth of 5 m	Isolation medium (yeast extract 0.25 g, K_2_HPO_4_ 0.5 g, agar 12 g, 500 mL seawater and 500 mL distilled water)	[132]
*Saccharopolyspora spongiae*/Pseudonocardiaceae	Marine sponge *Scopalina ruetzleri* at depths between 20 and 30 m	M1 medium [1% starch, 0.4% yeast extract, 0.2% peptone, 2% agar containing artificial seawater (33 g red sea salt L^−1^) amended with cycloheximide and nystatin (each at 25 µg mL^−1^)]	[40]
*Microbacterium aureliae*/Microbacteriaceae	Moon jellyfish *Aurelia aurita*	Zobell marine agar (HiMedia) and Tryptic soy agar (HiMedia)	[133]
*Mycobacterium stephanolepidis*/Mycobacteriaceae	Marine teleost fish *Stephanolepis cirrhifer*	Middlebrook 7H11 agar with oleic albumin dextrose catalase (OADC) enrichment (Becton Dickinson)	[134]
*Marmoricola aquaticus*/Nocardioidaceae	marine sponge *Glodia corticostylifera*	M1 agar (soluble starch 10 g L^−1^, yeast extract 4 g L^−1^, peptone 2 g L^−1^, agar 15 g L^−1^, 80% artificial seawater)	[38]
*Arthrobacter echini*/Micrococcaceae	Purple sea urchin *Heliocidaris crassispina*	Marine agar 2216 (Difco)	[135]
*Ornithinimicrobium algicola*/Intrasporangiaceae	Marine green alga *Ulva* sp.	Modified R2A medium (yeast extract 0.5 g, peptone 0.5 g, casein enzyme hydrolysate 0.5 g, yeast extract 0.5 g, glucose 0.5 g, water soluble starch 0.5 g, dipotassium phosphate 0.3 g, magnesium sulphate 0.05 g, sodium pyruvate 0.3 g, sodium chloride 20.0 g and distilled water 1000 mL)	[41]
*Nocardia xestospongiae*/Nocardiaceae	Marine sponge *Xestospongia* sp.	Modified starch-casein nitrate seawater agar containing 10 g soluble starch, 1 g sodium caseinate, 0.5 g KH_2_PO_4_, 0.5 g MgSO_4_ and 18 g agar in 1 L seawater, pH 8.3, supplemented with 50 mg nalidixic acid L^−1^ and 200 mg nystatin L^−1^	[136]
*Rubrobacter aplysinae*/Rubrobacteraceae	Marine sponge *Aplysina aerophoba*	Tryptone soy agar (Oxoid)	[137]
*Actinokineospora spheciospongiae*/Actinosynnemataceae	Marine sponge *Spheciospongia vagabunda*	ISP 2 medium (yeast extract 4.0 g, malt extract 10.0 g, dextrose 4.0 g, distilled water 1 L and Bacto agar 20.0 g)	[138]
*Williamsia spongiae*/Gordoniaceae	Marine sponge *Amphimedon viridis* at depths of between 5 and 10 m	Tryptic Soy Agar [Oxoid; prepared with 80% (v/v) artificial seawater]	[39]
*Myceligenerans cantabricum*/Promicromonosporaceae	Marine coral at a depth of 1500 m	1/3 Tryptic soy agar (Merck) and and 1/6 M-BLEB agar (9 g MOPS BLEB base (Oxoid) in 1 L Cantabrian seawater, containing the antifungal cycloheximide (80 µg mL^−1^) and anti-Gram-negative bacteria nalidixic acid (20 mg mL^−1^)	[139]

**Table 5:** New species of rare actinomycetes from mangrove environment reported during the period of mid 2013–2017.

Strain/Family	Nature of Sample	Isolation Medium	Ref.
*Lysinimicrobiumaestuarii*/Demequinaceae	Sediment of mangrove tidal flat	1/5 NBRC medium 802 [0.2% (w/v) polypeptone, 0.04% (w/v) yeast extract, 0.02% (w/v) MgSO_4_.7H_2_O and 1.5% (w/v) agar; pH 7.0] supplemented with 5.0% (w/v) NaCl, 0.005% (w/v) cycloheximide and 0.002% (w/v) nalidixic acid	[140]
*Lysinimicrobium flavum*/Demequinaceae	Rhizosphere soil of mangrove		
*Lysinimicrobium gelatinilyticum*/Demequinaceae			
*Lysinimicrobium iriomotense*/Demequinaceae			
*Lysinimicrobium luteum*/Demequinaceae	Soil of mangrove forest		
*Lysinimicrobium pelophilum*/Demequinaceae	Mud of mangrove tidal flat		
*Lysinimicrobium rhizosphaerae*/Demequinaceae	Rhizosphere soil of mangrove		
*Lysinimicrobium soli*/Demequinaceae	Soil of mangrove forest		
*Lysinimicrobium subtropicum*/Demequinaceae	Rhizosphere soil of mangrove		
*Micromonospora wenchangensis*/Micromonosporaceae	Mangrove soil	Glucose-peptone-tryptone agar supplemented with 50 mg nystatin L^−1^, 50 mg cycloheximide L^−1^, 25 mg novobiocin L^−1^ and 20 mg nalidixic acid L^−1^	[52]
*Micromonospora zhanjiangensis*/Micromonosporaceae	Mangrove soil	1/10 ATCC 172 agar supplemented with nalidixic acid (10 µg mL^−1^), novobiocin (10 µg mL^−1^), nystatin (50 µg mL^−1^) and K_2_Cr_2_O_7_ (20 µg mL^−1^)	[141]
*Micromonospora ovatispora*/Micromonosporaceae	Mangrove soil	ATCC 172 agar	[142]
*Micromonospora sediminis*/Micromonosporaceae	Mangrove sediment	AV medium (1.0 g glucose, 1.0 g glycerol, 0.3 g L-arginine, 0.3 g K_2_HPO_4_, 0.2 g MgSO_4_.7H_2_O, 0.3 g NaCl, 18 g agar, artificial seawater added up to 1 L)	[42]
*Micromonospora mangrovi*/Micromonosporaceae	Mangrove soil	Glucose-peptone-tryptone agar (glucose 10 g, peptone 5 g, tryptone 3 g, NaCl 5 g, agar 15 g, ddH_2_O 1 L supplemented with 50 mg/L of nystatin, 50 mg/L of cycloheximide, 25 mg/L of novobiocin and 20 mg/L of nalidixic acid)	[143]
*Nocardiopsis mangrovei*/Nocardiopsaceae	Mangrove sediment	Humic acid vitamin agar (humic acid 1.0 g, KCl 1.7 g, Na_2_HPO_4_ 0.5 g, MgSO_4_·7H_2_O 0.5 g, CaCO_3_ 0.02 g, FeSO_4_·7H_2_O, 0.01 g, B vitamins (0.5 mg each of thiamin, riboflavin, niacin, pyridoxin, calcium d-pantothenate, inositol, *p*-aminobenzoic acid and 0.25 mg biotin), cycloheximide 25 mg; potassium dichromate 50 mg, nystatin 50 mg, agar 15.0 g per litre of distilled water)	[27]
*Nocardiopsis sediminis*/Nocardiopsaceae	Mangrove sediment	Starch casein agar (1% soluble starch, 0.03% casein, 0.2% KNO_3_, 0.2% NaCl, 0.005% MgSO_4_.7H_2_O, 0.2% K_2_HPO_4_, 0.02% CaCO_3_, 0.001% FeSO_4_.7H_2_O, 1.8% agar)	[144]
*Sinomonas humi*/Micrococcaceae	Mangrove soil	Starch casein agar [1% soluble starch, 0.03% casein, 0.2% KNO_3_, 0.2% NaCl, 0.005% MgSO_4_.7H_2_O, 0.2% K_2_HPO_4_, 0.02% CaCO_3_, 0.001% FeSO_4_.7H_2_O, 1.8% agar supplemented with cycloheximide (25 µg mL^−1^) and nystatin (10 µg mL^−1^)]	[51]
*Kocuria pelophila*/Micrococcaceae	Rhizosphere soil of mangrove	NBRC medium 802 [1.0% (w/v) polypeptone, 0.2% (w/v) yeast extract, 0.1% (w/v) MgSO_4_.7H_2_O and 1.5% (w/v) agar]	[145]
*Mumia flava* (novel genus)/Nocardioidaceae	Mangrove soil	ISP 2 medium [yeast extract 4.0 g, malt extract 10.0 g, dextrose 4.0 g, Distilled water 1 L and Bacto agar 20.0 g supplemented with cycloheximide (25 µg mL^−1^) and nystatin (10 µg mL^−1^)]	[146]
*Monashia flava* (novel genus)/Intrasporangiaceae	Mangrove soil	Starch casein agar [1% soluble starch, 0.03% casein, 0.2% KNO_3_, 0.2% NaCl, 0.005% MgSO_4_.7H_2_O, 0.2% K_2_HPO_4_, 0.02% CaCO_3_, 0.001% FeSO_4_.7H_2_O, 1.8% agar supplemented with cycloheximide (25 µg mL^−1^) and nystatin (10 µg mL^−1^)]	[23]
*Pseudonocardia nematodicida*/Pseudonocardiaceae	Mangrove sediment	Modified gause inorganic agar (20 g soluble starch, 1 g KNO_3_, 0.5 g K_2_HPO_4_, 0.5 g MgSO_4_.7H_2_O, 0.01 g FeSO_4_.7H_2_O, 15 g agar, 1 L aged seawater)	[147]
*Microbacterium mangrovi*/Microbacteriaceae	Mangrove soil	Starch casein agar [1% soluble starch, 0.03% casein, 0.2% KNO_3_, 0.2% NaCl, 0.005% MgSO_4_.7H_2_O, 0.2% K_2_HPO_4_, 0.02% CaCO_3_, 0.001% FeSO_4_.7H_2_O, 1.8% agar supplemented with cycloheximide (25 µg mL^−1^) and nystatin (10 µg mL^−1^)]	[50]
*Actinoallomurus acanthiterrae*/Thermomonosporaceae	Rhizosphere soil of *Acanthus ilicifolius*	Oatmeal agar [Oatmeal 20.0 g, agar 18.0 g, supplemented with novobiocin (25 µg mL^−1^), nystatin (30 µg mL^−1^), nalidixic acid (10 µg mL^−1^) and K_2_Cr_2_O_7_ (20 mg mL^−1^]	[148]
*Jiangella mangrovi*/Jiangellaceae	Mangrove soil	Marine agar 2216 (Difco)	[149]
*Serinibacter tropicus*/Beutenbergiaceae	Rhizosphere soil of mangrove	NBRC medium 802 [0.2% (w/v) polypeptone, 0.04% (w/v) yeast extract, 0.02% (w/v) MgSO_4_.7H_2_O and 1.5% (w/v) agar] supplemented with 5.0% (w/v) NaCl, 0.005% (w/v) cycloheximide and 0.002% (w/v) nalidixic acid	[150]
*Nonomuraea purpurea*/Streptosporangiaceae	Mangrove sediment	Marine agar 2216 (Difco)	[151]
*Kineococcus mangrovi*/Kineosporiaceae	Mangrove sediment	Starch casein agar [1% soluble starch, 0.03% casein, 0.2% KNO_3_, 0.2% NaCl, 0.005% MgSO_4_.7H_2_O, 0.2% K_2_HPO_4_, 0.02% CaCO_3_, 0.001% FeSO_4_.7H_2_O, 1.8% agar supplemented with nalidixic acid (25 µg mL^−1^) and ketokonazole (100 µg mL^−1^)]	[152]

**Table 6:** Number of new species of rare actinomycetes reported from marine environment between 2007 and 2017.

Particular	2007 to mid-2013 *	Mid-2013 to 2017	Total (2007–2017)
New species reported	80	97	177
Novel families reported	3	-	3
Novel genera reported	20	9	29
Total families reported	23	27	33
**No. of new species reported in each family**			
Micromonosporaceae	13	10	23
Nocardioidaceae	10	9	19
Pseudonocardiaceae	6	11	17
Microbacteriaceae	5	8	13
Micrococcaceae	5	8	13
Demequinaceae	1	11	12
Nocardiopsaceae	4	6	10
Micrococcineae	10	-	10
Propionibacteriaceae	4	4	8
Intrasporangiaceae	4	4	8
Nocardiaceae	2	4	6
Streptosporangiaceae	3	1	4
Promicromonosporaceae	3	1	4
Cellulomonadaceae	1	2	3
Acidimicrobiaceae	1	2	3
Bogoriellaceae	1	2	3
Beutenbergiaceae	1	1	2
Thermomonosporaceae	1	1	2
Actinopolysporaceae	-	2	2
Brevibacteriaceae	-	2	2
Alteromonadaceae	1	-	1
Tsukamurellaceae	1	-	1
Iamiaceae	1	-	1
Euzebyaceae	1	-	1
Geodermatophilaceae	1	-	1
Actinomycetaceae	-	1	1
Rubrobacteraceae	-	1	1
Actinosynnemataceae	-	1	1
Gordoniaceae	-	1	1
Jiangellaceae	-	1	1
Kineosporiaceae	-	1	1
Dermacoccaceae	-	1	1
Dermabacteraceae	-	1	1

* Adapted from Subramani and Aalbersberg [7].

**Table 7:** Antibiotics of therapeutic natural products derived from actinomycetes.

Drug Name/Compound	Natural Product (NP) or Derivative	Source Organism	Chemical Class	Therapeutic Activity	Clinical Status (Year)	Reference
***Non-marine source***						
Telithromycin	Erythromycin (NP-derivative)	*Saccharopolyspora erythraea*	Macrolide	Antibacterial (G+ve/G−ve)	Approved (2001)	[158]
Biapenem	Thienamycin (NP-derivative)	*Streptomyces cattleya*	Carbapenem	Antibacterial (G+ve/G−ve)	Approved (2002)	[158]
Ertapenem	Thienamycin (NP-derivative)	*Streptomyces cattleya*	Carbapenem	Antibacterial (G+ve/G−ve)	Approved (2002)	[158]
Daptomycin	Natural product	*Streptomyces roseosporus*	Lipopeptide	Antibacterial (G+ve)	Approved (2003)	[158]
Doripenem	Thienamycin (NP-derivative)	*Streptomyces* sp.	Carbapenem	Antibacterial (G+ve/G−ve)	Approved (2005)	[158]
Tigecycline	Tetracycline (NP-derivative)	*Streptomyces aureofaciens*	Tetracycline	Antibacterial (G+ve/G−ve)	Approved (2005)	[158]
Tebipenem pivoxil	Thienamycin (NP-derivative)	*Streptomyces* sp.	Carbapenem	Antibacterial (G+ve/G−ve)	Approved (2009)	[158]
Telavancin	Vancomycin (NP-derivative)	*Amycolatopsis orientalis*	Glycopeptide	Antibacterial (G+ve)	Approved (2009)	[158]
Fidaxomicin	Natural product	*Dactylosporangium aurantiacum*	Tiacumicin	Antibacterial (G+ve)	Approved (2011)	[158]
Dalbavancin	Teicoplanin (NP-derivative)	*Nonomuria* sp.	Glycopeptide	Antibacterial (G+ve)	Approved (2014)	[158]
Oritavancin	Chloroeremomycin (NP-derivative)	*Amycolatopsis orientalis*	Glycopeptide	Antibacterial (G+ve)	Approved (2014)	[158]
Tazobactam	NP-derivative	Actinomycete strain	Penicillanic acid sulfone derivative and β-lactamase inhibitor	Antibacterial (G−ve)	Approved (2014)	[158]
***Marine source***						
Salinosporamide A (Marizomib)	Natural product	*Salinispora tropica*	Beta-lactone-gamma lactam	Multiple cancer	Phase I	[159]
Arenamides A and B	Natural product	*Salinispora* sp.	Peptide	Inflammation	Pre-clinical	[160]
Anthracimycin	Natural product	*Streptomyces* sp.	Polyketide	Anthrax	Pre-clinical	[161]

G+ve: Gram positive; G−ve: Gram negative.

**Table 8:** Numbers of new natural products/bioactive compounds produced by rare actinomycete genera from the marine environment between 2007 and 2017.

Particular	2007 to mid-2013 *	Mid-2013 to 2017	Total (2007–2017)
Novel/new compounds reported	100	167	267
Total number of rare actinomycetes	38	58	96
Total genera reported	15	24	28
**No. of new compounds reported in each genus**			
*Nocardiopsis*	12	40	52
*Micromonospora*	9	37	46
*Salinispora*	20	21	41
*Pseudonocardia*	3	14	17
*Verrucosispora*	18	2	20
*Amycolatopsis*	1	3	4
*Serinicoccus*	1	1	2
*Kocuria*	1	-	1
*Actinoalloteichus*	11	5	16
*Actinomadura*	3	5	8
*Dermacoccus*	7	3	10
*Kitasatospora*	1	-	1
*Nocardia*	2	-	2
*Saccharomonospora*	1	5	6
*Marinispora*	10	-	10
*Actinokineospora*	-	2	2
*Solwaraspora*	-	2	2
*Micrococcus*	-	1	1
*Microbacterium*	-	3	3
*Rubrobacter*	-	2	2
*Saccharothrix*	-	4	4
*Actinomycetospora*	-	3	3
*Williamsia*	-	1	1
*Streptomonospora*	-	4	4
*Nesterenkonia*	-	1	1
*Kribbella*	-	4	4
*Streptosporangium*	-	3	3
*Saccharopolyspora*	-	1	1

* Adapted from Subramani and Aalbersberg [7].

**Table 9:** Novel/new bioactive compounds produced by marine rare actinomycetes between mid 2013 and 2017.

Compounds	Chemical Family/Class	Marine Source	Biological Activity	Reference
Halomadurones A–D	Halogenated electrophilic pyrones	*Actinomadura* sp.	Potent Nrf2-ARE activation	[176]
Levantilide C	20-membered macrolide	*Micromonospora* sp.	Antiproliferative activity	[178]
Nocapyrones H–J	α-pyrones	*Nocardiopsis* sp.	Pro-inflammatory factor, stronger inhibitory effect on nitric oxide	[179]
Nocardiopsins C and D	Prolinyl-macrolactam polyketides	*Nocardiopsis* sp.	Not specified	[163]
Nocardiopyrone A	α-pyrone polyketide	*Nocardiopsis* sp.	Not specified	[163]
Nocardiamide A and B	Cyclic hexapeptides	*Nocardiopsis* sp.	Antimicrobial activity	[180]
Cyanosporasides C–F	Polyketides	*Salinispora pacifica*	Not specified	[181]
Micromonolactam	Polyene macrocyclic lactam	*Micromonospora* sp.	No antimicrobial activity	[165]
Cyanogramide	Spirocyclic alkaloid	*Actinoalloteichus cyanogriseus*	Multidrug-resistance (MDR) reversing activity	[177]
Actinosporins A and B	*O*-glycosylated angucyclines	*Actinokineospora* sp.	Moderate activity against *Trypanosoma brucei*	[182]
Solwaric acids A and B	Trialkyl-substituted aromatic acids	*Solwaraspora* sp.	Antibacterial activity against MDR pathogens	[183]
Seriniquinone	Quinones	*Serinicoccus* sp.	A selective anticancer agent	[184]
Cyanogrisides E–H	Acyclic bipyridine glycosides	*Actinoalloteichus cyanogriseus*	Cytotoxicity	[185]
Forazoline A	Polyketides	*Actinomadura* sp.	Anti-candida activity	[186]
Amycofuran	Benzofuran glycoside	*Amycolatopsis* sp.	Modest cytotoxicity	[175]
Amycolactam	Indole alkaloids	*Amycolatopsis* sp.	Cytotoxicity	[175]
Amycocyclopiazonic acid	Cyclopiazonic acid	*Amycolatopsis* sp.	Modest cytotoxicity	[175]
Dermacozines H–J	Heteroaromatic phenazines	*Dermacoccus abyssi*	Radical scavenging activity	[187]
Microluside A	*O*-glycosylated xanthone	*Micrococcus* sp.	Antibacterial activity	[188]
Nocapyrone R	α-pyrones	*Nocardiopsis* sp.	No cytotoxicity	[189]
Butremycin	Polycyclic tetramic acid macrolactams	*Micromonospora* sp.	Weak antibacterial activity	[167]
5′-Methylthioinosine	Protonated aromatic tautomer	*Micromonospora* sp.	No antibacterial activity	[167]
Butrepyrazinone	Pyrazinone	*Verrucosispora* sp.	No antibacterial activity	[172]
MBJ-0003	Hydroxamate	*Micromonospora* sp.	Moderate cytotoxicity	[190]
Microbacterins A and B	Peptaibols	*Microbacterium sediminis*	Potent cytotoxic activity	[191]
Salinipostins A–K	Bicyclic Phosphotriesters	*Salinospora* sp.	Antimalarial activity	[192]
Nocarimidazoles A and B	4-aminoimidazole alkaloids	*Nocardiopsis* sp.	Weak antibacterial activity	[162]
Dimeric indole derivatives 1 and 2	Dimeric indoles	*Rubrobacter radiotolerans*	Acetylcholinesterase (AchE) inhibitory activity	[174]
Saccharothrixones A–D	Aromatic polyketides	*Saccharothrix* sp.	Cytotoxic activity	[193]
Thiasporines A–C	Thiazine and Thiazole Derivatives	*Actinomycetospora chlora*	Cytotoxicity	[170]
Diketopiperazine 1	Diketopiperazine	*Nocardiopsis* sp.	Sterol *O*-acyltransferase inhibitor	[164]
Isopimara-2-one-3-ol-8,15-diene	Pimarane Diterpene	*Micromonospora* sp.	Weak cytotoxicity	[169]
Lagumycin B, Dehydrorabelomycin, Phenanthroviridone, WS-5995 A	Angucyclines	*Micromonospora* sp.	Cytotoxicity	[169]
Micromonohalimane A and B	Halimane-type diterpenoids	*Micromonospora* sp.	Modest antibacterial activity against MRSA, bacteriostatic	[168]
Quinoline alkaloid	Alkaloid	*Micromonospora* sp.	Antibacterial activity	[194]
1,4-dioxane derivative	Dioxane	*Micromonospora* sp.	Antibacterial activity	[194]
Pseudonocardides A–G	γ-butyrolactones	*Pseudonocardia* sp.	Antibacterial and cytotoxic activities	[195]
Curvularin macrolides 1–5	Macrolides	*Pseudonocardia* sp.	Antibacterial and cytotoxic activities	[171]
α-pyrones 1–8	α-pyrones	*Nocardiopsis* spp.	Moderate antibacterial activity	[196]
Compounds 1–12	Benzamides, Indoles	*Nocardiopsis* sp.	Antibacterial, antifungal and cytotoxic activities	[197]
3-benzyl-3α,4β-dihydroxypentan-2-one	Phenolics	*Williamsia* sp.	Not specified	[198]
Marinopyrones A–D	α-pyrones	*Streptomonospora* sp.	Inhibition of NO production	[199]
Glycerol 1-hydroxy-2,5-dimethyl benzoate	Salicylic derivative	*Verrucosispora* sp.	Anti-MRSA activity	[200]
Isomethoxyneihumicin	Lactam-lactim tautomers	*Nocardiopsis alba*	Strong cytotoxicity	[201]
Microindolinone A	Novel indole	*Microbacterium* sp.	No anti-allergic and anti-proliferative activities	[173]
Nesterenkoniane	Novel cyclic ether	*Nesterenkonia flava*	Moderate anti-allergic activity	[202]
Nocapyrones O–S	α-pyrones	*Nocardiopsis* sp.	Cytotoxicity	[203]
Paulomycin G	Glycosylated paulomycins	*Micromonospora matsumotoense*	Strong cytotoxic activity	[166]
Saccharomonopyrones A–C	α-pyrones	*Saccharomonospora* sp.	Weak antioxidant activity	[204]
Tetrocarcin Q	Spirotetronate glycoside	*Micromonospora carbonacea*	Moderate antibacterial activity	[205]
22-dehydroxymethyl-kijanolide	Spirotetronate aglycone	*Micromonospora harpali*	No antibacterial activity	[206]
8-hydroxy-22-dehydroxymethyl-kijanolide	Spirotetronate aglycone	*Micromonospora harpali*	No antibacterial activity	[206]
Microsporanates A–F	Spirotetronate glycosides	*Micromonospora harpali*	Antibacterial activity	[206]
Tetrocarcin P	Spirotetronate glycoside	*Micromonospora harpali*	Antibacterial activity	[206]
Nocazines F and G	Diketopiperazine	*Nocardiopsis* sp.	Excellent cytotoxicity	[207]
Kribellosides A-D	Alkyl glyceryl ethers	*Kribbella* sp.	Antifungal and RNA 5’-triphosphatase inhibitor	[208]
Branimycins B and C	Macrolide	*Pseudonocardia carboxydivorans*	Antibacterial activities	[209]
1,2-naphthoquinone	Naphthalene derivative	*Saccharopolyspora* sp.	No cytotoxicity	[210]

**Table 10:** New bioactive compounds discovered from marine rare actinomycetes using genome-based approaches between mid-2013 and 2017.

Compounds	Chemical Class/Family	Marine Source	Biological Activity	Reference
Taromycin A	Dichlorinated lipopeptide	*Saccharomonospora* sp.	Moderate bioactivity against MDR pathogens	[217]
Retimycin A	Quinomycin-like depsipeptide	*Salinispora* sp.	Cytotoxicity against HCT-116	[218]
Sioxanthin	Carotenoid	*Salinispora* sp.	Siderophore	[219]
Lobosamides A–C	Polyene macrolactams	*Micromonospora* sp.	Anti-protozoan parasite, *Trypanosoma brucei*	[220]
Hexaricins A–C	Pentangular polyphenols	*Streptosporangium* sp.	Not specified	[221]
Tetrocarcin N and O	Glycosidic spirotetronates	*Micromonospora* sp.	Modest antibacterial activity	[222]
Rifsaliniketal	Saliniketal	*Salinispora* sp.	Not specified	[223]
Nenestatin A	Benzofluorene	*Micromonospora echinospora*	Antibacterial activity	[224]
